# Systematic review of self‐management interventions for people with eczema

**DOI:** 10.1111/bjd.15601

**Published:** 2017-08-02

**Authors:** M.J. Ridd, A.J.L. King, E. Le Roux, A. Waldecker, A.L. Huntley

**Affiliations:** ^1^ School of Social and Community Medicine University of Bristol Bristol U.K

## Abstract

Eczema is a common long‐term condition, but inadequate support and information can lead to poor adherence and treatment failure. We have reviewed the international literature of interventions designed to promote self‐management in adults and children with eczema. MEDLINE, MEDLINE in process, Embase, CINAHL and the Global Resource for EczemA Trials database were searched from their inception to August 2016, for randomized controlled trials. Two authors independently applied eligibility criteria, assessed risk of bias for all included studies and extracted data. Twenty studies (3028 participants) conducted in 11 different countries were included. The majority (*n *=* *18) were based in secondary care and most (*n *=* *16) targeted children with eczema. Reporting of studies, including descriptions of the interventions and the outcomes themselves, was generally poor. Thirteen studies were face‐to‐face educational interventions, five were delivered online and two were studies of written action plans. Follow‐up in most studies (*n *=* *12) was short term (up to 12 weeks). Only six trials specified a single primary outcome. There was limited evidence of effectiveness. Only three studies collected and reported outcomes related to cost and just one study undertook any formal cost‐effectiveness analysis. In summary, we have identified a general absence of well‐conducted and well‐reported randomized controlled trials with a strong theoretical basis. Therefore, there is still uncertainty about how best to support self‐management of eczema in a clinically effective and cost‐effective way. Recommendations on design and conduct of future trials are presented.

## Abstract 2

**What's already known about this topic?**

- Eczema requires a high degree of self‐management by patients.
- Adherence to eczema treatments, and hence control of symptoms, can be poor.
- There is uncertainty about how best to support self‐management in a clinically effective and cost‐effective way.

**What does this study add?**

- A wide range of interventions designed to promote self‐management have been evaluated in 20 studies across 11 different countries.
- Reporting of the design and conduct of these studies is generally poor, and explicit theory describing how interventions are expected to improve care is uncommon.
- What works best for people with eczema and whether it is cost‐effective is unknown.
- Recommendations for future trials are made.

**Linked Comment:** Arents. *Br J Dermatol* 2017; **177**:613–614

Plain language summary available online

Eczema is a long‐term condition that usually begins in infancy and can have a significant impact on patient quality of life. Also referred to as atopic dermatitis and atopic eczema, the World Allergy Organization suggests that the phenotype of ‘atopic eczema’ should be simply called ‘eczema’ unless specific IgE antibodies are demonstrated.^1^ Eczema is common and its prevalence is increasing. Approximately 20% of children in industrialized countries have eczema, and in developing countries the prevalence is heading towards this figure.^2^ In adults, population studies report an overall prevalence of 2–18%.^3^ It is also a condition for which a high degree of self‐care is needed.^4^

Recently, there has been a policy shift in the U.K. towards self‐management for long‐term conditions. Interventions to improve patient (or carer) self‐management of long‐term conditions are broadly designed to ‘increase the capacity, confidence and efficacy of the individual’ to manage their health on a day‐to‐day basis.^5^ Improved self‐management has been identified as key in improving disease outcomes and promoting quality of life for people with long‐term conditions.^6^ Effective treatment of eczema demands good self‐management, which, if established early on, could lead to considerable improvement in quality of life. However, families of children with eczema state that they do not receive adequate support and information about symptom management.^4^ A lack of education about therapy can lead to poor adherence (patients/carers not using creams effectively) and treatment failure.^7^

In view of this, we sought to review the evidence on the effectiveness of interventions designed to promote self‐management for children, their caregivers and adults with eczema. In particular, we wanted to answer the following questions: What evidence is there that interventions designed to promote self‐management of atopic eczema are clinically effective and cost‐effective? What have the interventions evaluated to date comprised? Has previous research established the contribution of the different components of self‐management interventions to the outcomes assessed?

## Materials and methods

We followed Preferred Reporting Items for Systematic reviews and Meta‐Analyses (PRISMA) guidelines^8^ and the protocol was prospectively registered with PROSPERO (PROSPERO 2015:CRD42015025314).^9^

### Information sources and search strategy

We searched relevant databases (MEDLINE, MEDLINE In Process, Embase, CINAHL and Global Resource for EczemA Trials,^10^ from inception to August 2016) for randomized controlled trials (RCTs) of interventions (delivered to children with eczema, caregivers of children with eczema and adults with eczema) that promote self‐management. With the aid of a medical information scientist, a search strategy was developed that included the following terms: eczema (and its synonyms atopic eczema and atopic dermatitis), self‐care, self‐management, education, patient education, action plan, treatment plan and management plan (Appendix S1; see Supporting Information). Authors were contacted regarding further trial publications and any unpublished studies and/or unpublished data. Forward and backward searching was also conducted within the reference lists of all included studies.

References from the searches were downloaded into Endnote (Endnote X7, Thomson Reuters, New York, NY, U.S.A.). Two people independently screened all titles and abstracts using the eligibility criteria. All included studies were accessed in full and were screened by two reviewers independently. The reasons for exclusion of all full‐text trials were recorded and any disagreements were resolved by the research team.

### Eligibility criteria

We restricted our search to RCTs of interventions that promote patient/carer self‐management in children (and/or their caregivers, including parents) and adults with atopic eczema/atopic dermatitis, compared with no intervention, usual care, or an alternative intervention. The outcomes of primary interest were effects on eczema severity and quality of life.

There is no agreed definition of self‐management. Therefore, based on the relevant literature,^11^, ^12^, ^13^, ^14^ we defined a self‐management intervention as one that included one or more of the features listed in Table 1. If a trial included patients with other skin diseases, and the data for eczema could not be analysed separately, it was excluded. As our main outcomes of interest were eczema severity and quality of life, we excluded trials that did not include these outcomes.

**Table 1:** Definition of interventions that promote patient/carer self‐management

Imparts knowledge of the condition and/or its management Supports people in managing the social, emotional or physical impacts of their conditions Involves patients/carers in decision‐making Motivates people to self‐manage (using targeted approaches and/or structured support) Helps people to monitor their symptoms and know when to take appropriate action, for example through the use of written action plans

### Data extraction and risk of bias

A data extraction tool was developed and piloted. Data on study design, description of intervention/comparison components and outcomes were extracted independently, and in duplicate, by two reviewers (A.J.L.K./E.L.R. and M.J.R.). Authors were contacted to confirm missing data. Risk of bias was conducted by two blinded reviewers (A.J.L.K./E.L.R.) and checked by a third (M.J.R.), using the Cochrane Collaboration's risk of bias tool^15^ and Review Manager software (version 5·3, Informer Technologies Inc., Roseau, Dominica).

## Results

### Study selection

**Figure 1:**
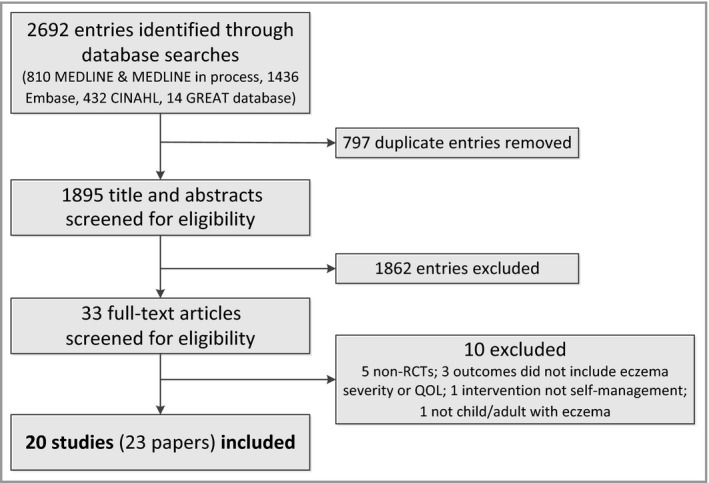
Flowchart showing the flow of studies through the systematic review.

After deduplication, 1895 titles and abstracts were screened for eligibility and 33 full‐text papers were assessed for eligibility (Fig. 1). After the exclusion of 10 papers, we included 23 articles^16^, ^17^, ^18^, ^19^, ^20^, ^21^, ^22^, ^23^, ^24^, ^25^, ^26^, ^27^, ^28^, ^29^, ^30^, ^31^, ^32^, ^33^, ^34^, ^35^, ^36^, ^37^, ^38^ that described 20 RCTs. Two studies were published in German^25^, ^27^ and were translated for the purpose of this review. Two articles were published as research letters.^23^, ^32^

### Design, setting and participant recruitment

Participants were individually randomized in all 20 studies, which included a total of 3028 participants (Tables 2 and 3). The majority of studies were conducted in Europe (*n *=* *14) and the U.S.A. (*n *=* *4). Most (*n *=* *18) were set in secondary care with participants recruited via dermatology^16^, ^17^, ^19^, ^20^, ^23^, ^24^, ^25^, ^26^, ^27^, ^28^, ^30^, ^32^, ^38^ and paediatric outpatient clinics.^22^, ^31^, ^34^, ^35^, ^37^

**Table 2:** Summary of included studies

Characteristic	Number of studies	Study (First author, year)
Country		
U.S.A.	4	Armstrong *et al*. 2011, Shaw *et al*. 2008, Shi *et al*. 2013, Gilliam *et al*. 2016
Germany	4	Kardorff *et al*. 2003, Niebel *et al*. 2000, Staab *et al*. 2002, Staab *et al*. 2006
U.K.	2	Chinn *et al*. 2002, Santer *et al*. 2014
The Netherlands	2	Schuttelaar *et al*. 2009, van Os‐Medendorp *et al*. 2012
Australia	2	Grillo *et al*. 2006, Moore *et al*. 2009
Norway	1	Bergmo *et al*. 2008
Belgium	1	Bostoen *et al*. 2012
Croatia	1	Pustišek *et al*. 2016
Sweden	1	Broberg *et al*. 1990
Japan	1	Futamura *et al*. 2013
Republic of Korea	1	Son *et al*. 2014
Setting		
Secondary care	18	Armstrong *et al*. 2011, Bergmo *et al*. 2008, Bostoen *et al*. 2012, Broberg *et al*. 1990, Futamura *et al*. 2013, Gilliam *et al*. 2016, Grillo *et al*. 2006, Kardorff *et al*. 2003, Moore *et al*. 2009, Niebel *et al*. 2000, Pustišek *et al*. 2016, Schuttelaar *et al*. 2009, Shaw *et al*. 2008, Shi *et al*. 2013, Staab *et al*. 2002, Staab *et al*. 2006, Son *et al*. 2014, van Os‐Medendorp *et al*. 2012
Primary care	2	Chinn *et al*. 2002, Santer *et al*. 2014
Participants		
Children only	16	Bergmo *et al*. 2008, Broberg *et al*. 1990, Chinn *et al*. 2002, Futamura *et al*. 2013, Gilliam *et al*. 2016, Grillo *et al*. 2006, Kardorff *et al*. 2003, Moore *et al*. 2009, Niebel *et al*. 2000, Pustišek *et al*. 2016, Santer *et al*. 2014, Staab *et al*. 2002, Son *et al*. 2014, Schuttelaar *et al*. 2009, Shaw *et al*. 2008, Staab *et al*. 2006
Adults only	2	Armstrong *et al*. 2011, Bostoen *et al*. 2012
Adults and children	2	Shi *et al*. 2013, van Os‐Medendorp *et al*. 2012
Inclusion criteria		
Eczema diagnosis		
Not stated	12	Bergmo *et al*. 2008, Bostoen *et al*. 2012, Futamura *et al*. 2013, Gilliam *et al*. 2016, Grillo *et al*. 2006, Kardorff *et al*. 2003, Moore *et al*. 2009, Niebel *et al*. 2000, Shaw *et al*. 2008, Shi *et al*. 2013, Son *et al*. 2014, van Os‐Medendorp *et al*. 2012
Hanifin and Rajka	5	Armstrong *et al*. 2011, Broberg *et al*. 1990, Pustišek *et al*. 2016, Staab *et al*. 2002, Staab *et al*. 2006
U.K. diagnostic criteria	2	Chinn *et al*. 2002, Schuttelaar *et al*. 2009
Clinical (GP diagnosis)	1	Santer *et al*. 2014
Minimum eczema severity		
None	13	Armstrong *et al*. 2011, Bostoen *et al*. 2012, Broberg *et al*. 1990, Chinn *et al*. 2002, Gilliam *et al*. 2016, Grillo *et al*. 2006, Moore *et al*. 2009, Niebel *et al*. 2000, Santer *et al*. 2014, Schuttelaar *et al*. 2009, Shaw *et al*. 2008, Shi *et al*. 2013, Son *et al*. 2014
Moderate	1	van Os‐Medendorp *et al*. 2012 (not defined)
Moderate‐to‐severe	6	Bergmo *et al*. 2008 (not defined), Futamura *et al*. 2013 (not defined), Kardorff *et al*. 2003 (SCORAD of between 25 and 50), Pustišek *et al*. 2016 (SCORAD > 25), Staab *et al*. 2002 [(SCORAD > 20) for at least 4 months], Staab *et al*. 2006 (SCORAD ≥ 20)
Duration of follow‐up		
Not stated	1	Shi *et al*. 2013
2 weeks	1	Son *et al*. 2014
4 weeks	1	Moore *et al*. 2009
6 weeks	1	Kardorff *et al*. 2003
1–3 months	1	Shaw *et al*. 2008
2 months	1	Pustišek *et al*. 2016
12 weeks/3 months	6	Armstrong *et al*. 2011, Broberg *et al*. 1990, Chinn *et al*. 2002, Gilliam *et al*. 2016, Grillo *et al*. 2006, Santer *et al*. 2014
3–4 months	1	Niebel *et al*. 2000
6 months	1	Futamura *et al*. 2013
9 months	1	Bostoen *et al*. 2012
12 months	5	Bergmo *et al*. 2008, Schuttelaar *et al*. 2009, Staab *et al*. 2002, Staab *et al*. 2006, van Os‐Mendendorp *et al*. 2012
Primary outcome^a^		
Not specified	10	Bergmo *et al*. 2008, Broberg *et al*. 1990, Chinn *et al*. 2002, Gilliam *et al*. 2016, Kardorff *et al*. 2003, Niebel *et al*. 2000, Shaw *et al*. 2008, Shi *et al*. 2013, Son *et al*. 2014, Staab *et al*. 2002
POEM	2	Armstrong *et al*. 2011, Santer *et al*. 2014
PO‐SCORAD	1	Pustišek *et al*. 2016
SCORAD	6	Futamura *et al*. 2013, Grillo *et al*. 2006, Moore *et al*. 2009, Staab *et al*. 2006, Pustišek *et al*. 2016, Bostoen *et al*. 2012
EASI	1	Bostoen *et al*. 2012
IDQOL/CDLQI	2	Schuttelaar *et al*. 2009, van Os‐Medendorp *et al*. 2012
DLQI	2	van Os‐Medendorp *et al*. 2012, Bostoen *et al*. 2012
Skindex‐29	1	Bostoen *et al*. 2012
QoLIAD	1	Bostoen *et al*. 2012
‘Quality of life in parents of children with atopic eczema questionnaire’	1	Staab *et al*. 2006

POEM, Patient‐Oriented Eczema Measure; SCORAD, SCORing Atopic Dermatitis; PO‐SCORAD, Patient‐Oriented SCORAD; EASI, Eczema Area and Severity Index; IDQOL, Infant's Dermatitis Quality of Life Index; CDLQI, Children's Dermatology Life Quality Index; DLQI, Dermatology Life Quality Index. QoLIAD, Quality of Life Index for Atopic Dermatitis. ^a^Four studies [Staab *et al*. (2006),^35^ van Os‐Medendorp *et al*.,^38^ Pustišek *et al*.^28^ and Bostoen *et al*.]^19^ specified multiple primary outcomes, therefore the column for this section does not add up to a total of 20.

**Table 3:** Details of included studies

Study no.	First author, year	Country	Setting	Total number of participants	Participants	Intervention	Comparison	Outcomes
1	Armstrong 2011^16^	U.S.A	Secondary care	80	Adults with atopic dermatitis	Online video‐based patient education that aims to improve atopic dermatitis knowledge and disease severity	Identical information in written pamphlet form	Primary: POEM
								Secondary: knowledge of atopic dermatitis and skincare for atopic dermatitis questionnaire; satisfaction with education material
2	Bergmo 2009^17^	Norway	Secondary care	98	Parents of children with atopic eczema	Web‐based consultations for parents of children with atopic dermatitis	Usual care (seek treatment through general practitioner visits and hospital care)	Primary outcome not specified
								Self‐management behaviour – number/frequency of skincare treatments performed by parent/carer
								Eczema severity (SCORAD)
								Resource use (self‐report of number of general practitioner visits, outpatient consultations, emergency visits, hospital admissions, visits to complementary therapists)
								Personal expenses and loss of employment
								Family costs
3	Bostoen 2012^18^, ^19^	Belgium	Secondary care and patient advocacy groups	50	Adults with atopic dermatitis or psoriasis	Educational programme (described in Lambert *et al*. 2011) 18 h twice weekly for 12 weeks	Not stated	Primary outcomes: SCORAD, EASI, DLQI, Skindex‐29 and QoLIAD
						Multidisciplinary educational programme delivered by a dermatologist, dermatology nurse, pharmacist, psychiatrist, psychologist, dietician, philosopher, mindfulness and yoga teacher		Secondary outcomes: Beck Depression Inventory, smoking behaviour, physical activity and everyday problem checklist (stress), EQ5‐D
						Content of programme: specific information on skin diseases; stress‐reduction techniques; information sessions on lifestyle factors and psychodermatology		Costs: topical (corticosteroids, calcineurin inhibitors, hydration) and systemic therapy; medication and doctor visits related to skin disease
4	Broberg *et al*. 1990^20^	Sweden	Secondary care	50	Children with atopic eczema	‘Eczema school’, health education intervention aimed at parents of children with atopic eczema. This was run for patients by a trained nurse who offered practical training on management of atopic eczema	Control group received routine information given by the physician during a medical visit	Primary outcome not specified. Physician‐assessed ‘Eczema score’ of 0–96 (based on type, intensity and distribution of lesions), itch score (0 = none to 4 = severe); topical steroid use (by weight)
5	Chinn 2002^21^	U.K.	Primary care	240	Children with atopic eczema	Patient education provided by a primary care nurse in a single (30 min) consultation	Usual care	Primary outcome not specified
								Quality of life (IDQOL or CDLQI; Family Dermatitis Index)
6	Futamura 2013^22^	Japan	Secondary care	59	Children with atopic eczema and their parents	2‐day Parental Education Programme on managing childhood eczema. Parents received this along with a booklet on atopic dermatitis	Parents were given a booklet about atopic dermatitis and received usual care	Primary: SCORAD
								Secondary: objective SCORAD; symptom scores (0–10) for pruritus and sleeplessness; corticosteroid cream use (total weight estimated by counting no tubes used), Dermatitis Family Impact questionnaire, parental anxiety about topical corticosteroid use
7	Gilliam 2016^23^	U.S.A.	Secondary care	88	Parents of children with atopic eczema	Eczema Action Plan	Standard clinical care/education	Childhood Eczema Study Questionnaire score (derived from Childhood AD Impact Score, Chamlin *et al*.)^55^
8	Grillo 2006^24^	Australia	Secondary care	61	Children with atopic eczema and their parents	2‐h group workshop	Usual care (routine education, medical consultation and management)	Primary outcome not specified. Eczema severity (SCORAD); Quality of life: CDLQI, IDQoL and Dermatitis Family Impact questionnaire
9	Kardorff 2003^39^	Germany	Secondary care	30	Caregivers (parents) of/ children with atopic eczema	10‐min consultation with a dermatologist including routine explanation of diagnosis and treatment plus ‘Hautmodell’ (skin model) – a 3D educational tool, developed by the study authors, which demonstrated to children and their parents the rationale behind regular emollient use	10‐min consultation with a dermatologist including routine explanation of diagnosis and treatment plus verbal instruction on emollient application (as per routine dermatological practice)	Eczema severity of children (SCORAD)
								Change in emollient use by parents
10	Moore 2009^26^	Australia	Secondary care	165	Children and adolescents with atopic eczema	Nurse‐led eczema workshop	A dermatologist‐led clinic (registrar or consultant)	Primary outcome: SCORAD
								Secondary outcome: comparison of eczema treatments used by patients in the eczema workshop and in the dermatologist‐led clinic
11	Niebel 2000^27^	Germany	Secondary care	47	Children with eczema and their mothers	Behaviour‐based parental education (direct parent education, 10 sessions) or video education at home	Usual care	Skin condition
								Symptomatic behaviour of the children with atopic eczema
								Problems faced by mothers and the burden they experience
12	Pustišek 2016^28^	Croatia	Secondary care	134	Parents of children with atopic eczema	2‐h structured educational programme comprising a lecture by dermatologist and nurse, written material including educational booklet and a diary of corticosteroid use	Usual care	SCORAD, PO‐SCORAD, pruritus symptoms score, sleeplessness symptoms score, Perceived Stress Scale, State Trait Anxiety Inventory, Croatian version of Family Dermatology Life Quality Index, use of topical corticosteroids (not clear how collected)
13	Santer 2014^29^	U.K.	Primary care	143	Children with eczema and their parents/carers	Website intervention only or website plus healthcare professional support	Usual care	Primary outcome: Eczema severity (POEM)
								Secondary outcomes: Quality of life (DFI, DQoL and CDLQI, Secondary outcome: adherence to interventions (Problematic Experiences of Therapy Scale)
14	Schuttelaar 2010^30^	The Netherlands	Secondary care	160	Children with eczema	Nurse practitioner (NP) routinely followed up 2 weeks after the first visit. Thereafter, the visits depended on the severity of the eczema and the needs of the parents. Average visit length: first, 30 min, second 10 min (telephone) or 20 min (clinic). It was possible to contact the NP for feedback, support or tips by mail and telephone daily. The NP was supervised by an independent dermatologist if necessary. Parents were also provided with a Written Eczema Action Plan. Information and instruction were offered during the treatment visits or in a 2‐h group session comprising a maximum of eight parents	Usual care (dermatologist). Number and interval between the treatment visits depended on the severity of the eczema. Average visit length: first 20 min, second 10 min, ± 5‐min telephone call for laboratory results on allergy tests. Patients received no education from the nurse	Primary outcomes: change in quality of life at 12 months (IDQOL) for children under 4 years and CDLQI for children aged 4–16 years
								Secondary outcomes: changes in IDQOL and CDLQI at 4 and 8 months postintervention. Family impact of eczema (Dermatitis Family Impact Questionnaire), eczema severity (SCORAD)
15	Shaw 2008^31^	U.S.A.	Secondary care	151	Children with eczema	5‐min individual face‐to‐face education session with an atopic dermatitis educator	Usual care, an individual treatment plan for each child was verbally explained to the family with some written notes given if deemed necessary	Primary outcome: eczema severity (SCORAD)
								Secondary outcome: change in infant's quality of life (IDQOL)
								Change in children's quality of life (CDLQI)
16	Shi 2013^32^	U.S.A.	Secondary care	37	Adults and children with atopic eczema	Eczema Action Plan	Verbal instruction only	Participants’ understanding of their individualized treatment plan, benefits and risks of the prescribed medication, anatomic location of medication use, duration of treatment, recognizing exacerbating factors, adjusting treatment based on disease severity, comfort about their treatment plan, anxiety about caring for atopic eczema at home, understanding of atopic eczema and ability to recognize disease remission
17	Staab 2002^33^, ^34^	Germany	Secondary care	204	Children with eczema	Structured parental training programme on managing atopic dermatitis in children (six group sessions, 2 h each)	Usual care (routine information from the physician during a medical visit). The control group could participate in the parental training programme 1 year after the randomized controlled trial	Primary outcome not specified. Eczema severity (SCORAD), treatment behaviours, treatment costs, quality of life [disease specific (quality of life in parents of children with atopic dermatitis) and generic (‘daily life’)] and coping strategies (The Trier Scales of Coping)
18	Staab 2006^35^, ^36^	Germany	Secondary care	992	Children (aged 3 months to 7 years, 8–12 years) and adolescents (13–18 years) with eczema	Parent/patient education sessions, different for each of the three age groups	Usual care	Primary outcomes: eczema severity (SCORAD) and parents’ quality of life (‘quality of life in parents of children with atopic dermatitis’)
								Secondary outcomes: subjective severity score (skin detective), itch (catastrophization and coping, measured using JUCKKI and JUCKJU)
19	Son 2014^37^	Republic of Korea	Secondary care	40	Parents of children with atopic eczema	Web‐Based Educational Programme	Not stated	Primary outcome not specified
								Parent‐reported global eczema severity, area of lesion and treatment method. Korean language versions of POEM, IDQoL and Child Eczema Management Questionnaire
								Mothers’ self‐efficacy
20	van Os‐Medendorp 2012^38^	The Netherlands	Secondary care	199	Adults and children with atopic dermatitis	Dermatologist and dermatology nurse outpatient appointment followed by dermatologist appointment 6 weeks later and no further scheduled appointments. An internet‐guided monitoring and online self‐management training intervention, which included patient‐initiated access to an eczema portal. Face‐to‐face visits to the dermatology nurse or dermatologist were possible in individual cases where e‐health was inadequate or when requested by the patients	Dermatologist and dermatology nurse outpatient appointment. After that, usual care (five scheduled follow‐up visits to the dermatologist, and at least one visit to a dermatology nurse for self‐management training depending on disease severity)	Primary outcomes: quality of life (DLQI for adults and IDQOL for infants), eczema severity
								Secondary outcomes: direct and indirect costs of care, costs of e‐health service, outpatient visits and days taken off work by adult patients and parents of children with atopic dermatitis

POEM, Patient‐Oriented Eczema Measure; SCORAD, SCORing Atopic Dermatitis; PO‐SCORAD, patient‐oriented SCORAD; EASI, Eczema Area and Severity Index; IDQOL, Infant's Dermatitis Quality of Life Index; CDLQI, Children's Dermatology Life Quality Index; DLQI, Dermatology Life Quality Index; QoLIAD, Quality of Life Index for Atopic Dermatitis; EQ5‐D, EuroQol five dimensions questionnaire; JUCKKI, Juckreiz‐Kognitions‐Fragebogen Kinder; JUCKJU, Juckreiz‐Kognitions‐Fragebogen Jugendliche.

With the exception of two three‐group trials,^27^, ^29^ most studies comprised two groups (intervention and comparison groups). Only five studies^27^, ^29^, ^34^, ^35^, ^37^ gave details of any theoretical framework that underpinned intervention development or possible mechanisms of effect.

### Characteristics of participants

In the majority of studies (*n *=* *16), the participants with eczema were children, but two studies were of adults with eczema,^16^, ^19^ and two were of adults and children with eczema.^32^, ^38^ One study included participants with eczema, psoriasis and other chronic skin diseases.^19^

Regarding inclusion criteria (Table 2), two studies stated that participants had to have been diagnosed for at least 3 months^28^, ^35^ and two studies specified 1 year.^29^, ^37^ Twelve studies did not specify any diagnostic criteria,^23^ and seven studies included only participants with moderate^38^ or moderate‐to‐severe eczema,^17^, ^22^, ^25^, ^28^, ^34^, ^35^ although how this was determined was not clear in three studies.^17^, ^22^, ^38^ In most studies of children, the caregivers were parents (three studies specify mothers),^22^, ^27^, ^37^ but in six studies the ‘caregiver’ was not further described.^23^, ^29^, ^30^, ^31^, ^32^, ^35^ Broberg *et al*.^20^ did not report participant baseline characteristics.

### Interventions and comparison groups

Of the studies aiming to improve the self‐management of  eczema in children, only the caregivers of children with eczema were the recipients of the intervention in eight studies,^17^, ^22^, ^23^, ^26^, ^27^, ^28^, ^29^, ^31^, ^34^, ^37^ while eight studies included children with eczema and their caregiver.^20^, ^21^, ^24^, ^25^, ^30^, ^35^ However, this distinction was often not very well described.

The majority of interventions (13 studies) were face‐to‐face educational interventions.^19^, ^20^, ^21^, ^22^, ^24^, ^25^, ^26^, ^27^, ^28^, ^30^, ^31^, ^34^, ^35^ Seven face‐to‐face educational interventions were delivered to groups,^19^, ^20^, ^22^, ^24^, ^28^, ^34^, ^35^ four were delivered to individuals,^21^, ^25^, ^30^, ^31^ and two to a mixture of individuals and groups.^26^, ^27^ In one study, three different variations of intervention were delivered according to the age of the child (3 months to 7 years, 8–12 years and 13–18 years).^35^ The duration and intensity of interventions varied from a one‐off 15‐min educational session,^31^ to 12 weekly 2‐h sessions.^19^ Interventions were delivered by between one and four health professionals including dermatologists, specialist dermatology nurses, nurse practitioners and interdisciplinary teams. Eighteen studies gave details on the type of health professional delivering the intervention,^16^, ^17^, ^19^, ^20^, ^21^, ^22^, ^25^, ^26^, ^27^, ^28^, ^29^, ^30^, ^31^, ^32^, ^34^, ^35^, ^37^, ^38^ three stated the level of staff training^16^, ^21^, ^31^ and 13 studies^16^, ^19^, ^20^, ^21^, ^22^, ^26^, ^28^, ^29^, ^30^, ^31^, ^34^, ^35^, ^38^ stated the number of health professionals that were involved in delivering the intervention. Most studies of face‐to‐face education (*n *=* *11) compared their intervention with ‘usual care’,^20^, ^21^, ^24^, ^25^, ^26^, ^27^, ^28^, ^30^, ^31^, ^34^, ^35^ although this was often not made explicit and/or the specific details of usual care were unclear.

Five studies were delivered via the internet,^16^, ^17^, ^29^, ^37^, ^38^ of which three studies compared their intervention with ‘usual care’.^17^, ^29^, ^38^ These varied from simple online videos^16^ and educational modules^29^, ^37^ to online consultations.^17^, ^38^ The study by Santer *et al*.^29^ was a pilot trial that included a third group in which healthcare professionals familiarized themselves with the intervention and, in a 20‐min appointment, encouraged participants to use the website as a resource to help them manage their child's eczema.

A written action plan was the intervention itself in two studies,^23^, ^32^ and was included as part of the educational package in a third.^30^

### Duration of follow‐up and collection of outcomes

Follow‐up in most studies was between 2 weeks and 12 weeks.^16^, ^20^, ^21^, ^23^, ^24^, ^26^, ^28^, ^29^, ^31^, ^37^, ^39^ Seven studies included longer follow‐up periods of 6 months,^22^ 9 months^19^ and 12 months.^17^, ^30^, ^34^, ^35^, ^38^ One study did not state the duration of follow‐up^32^ and two studies^27^, ^31^ stated varying follow‐up intervals.

### Outcomes

Only six of the 20 trials specified a single primary outcome (Table 2).^16^, ^22^, ^24^, ^26^, ^29^, ^30^ The studies by Staab *et al*. (2006),^35^ van Os‐Medendorp *et al*.^38^ Pustišek *et al*.^28^ and Bostoen *et al*.^19^ specified two, three, four and five primary outcomes, respectively. No primary outcome was specified in the other 10 studies.^17^, ^20^, ^21^, ^23^, ^25^, ^27^, ^31^, ^32^, ^34^, ^37^

A wide range of other/secondary outcomes were also collected, often using modified versions of published questionnaires, or unpublished and unvalidated scales. Only the following three studies collected and reported any outcomes related to cost: Staab *et al*. (2002)^34^ reported direct costs of treatment; Bergmo *et al*.^17^ reported loss of employment; Bergmo *et al*.^17^ and Bostoen *et al*.^19^ reported cost of contact with healthcare professionals and prescription costs; and van Os‐Medendorp^38^ reported direct and indirect participant costs. Only Bostoen *et al*.^19^ reported undertaking a formal cost‐effectiveness (cost‐utility) analysis, and simply concluded that their intervention (2‐h group‐based educational sessions per week for 12 weeks) was not cost‐effective at 6 months. It does not appear that separate analyses were done for the 21 of 59 participants with atopic eczema in this study.

**Figure 2:**
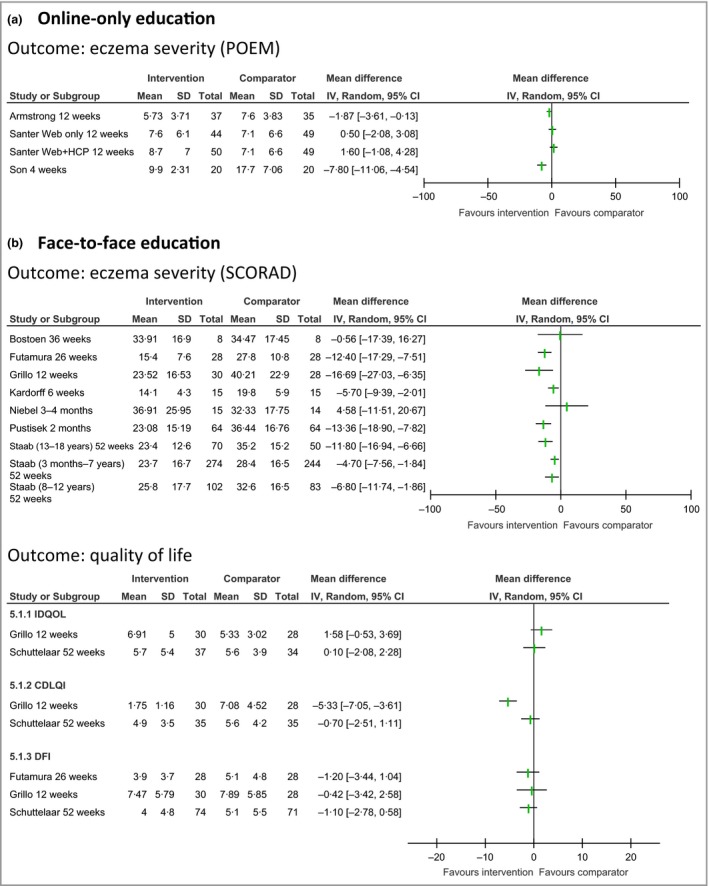
Forest plot of outcomes by intervention type. (a) Online‐only education. (b) Face‐to‐face education. POEM, Patient‐Oriented Eczema Measure; CI, confidence interval; HCP, healthcare professional; SCORAD, SCORing Atopic Dermatitis; IDQOL, Infant's Dermatitis Quality of Life Index; CDLQI, Children's Dermatology Life Quality Index; DFI, Dermatitis Family Impact Questionnaire.

We did not attempt to perform a meta‐analysis because there were not at least three similar studies with a low risk of bias. In addition, data on outcomes (e.g. means, SDs) on eczema severity and quality‐of‐life outcomes were often not reported (a complete list is presented in Appendix S2 and Appendix S3; see Supporting Information). We have summarized the findings graphically in Figure 2 for these outcomes where reported by two or more studies.

The available evidence suggests that the interventions developed and evaluated to date may improve both patient‐reported and objective measures of eczema severity but not quality of life. The three web‐based studies^16^, ^29^, ^37^ report changes in Patient‐Oriented Eczema Measure scores at 4–12 weeks near or greater than the published minimal clinically important difference (MCID) of 3.^40^ However, the studies themselves are quite different. Armstrong *et al*.^16^ compared an educational video with an information leaflet for adults recruited from a U.S. dermatology clinic; Santer *et al*.^29^ compared an educational website (with or without healthcare professional support) with usual U.K. primary care for caregivers of children under 5 years and Son *et al*.^37^ recruited parents of children in Korea under 3 years of age via a paediatric clinic to use a website, but did not describe what participants in their control group received.

The face‐to‐face interventions trialled by Futamura *et al*.,^22^ Grillo *et al*.,^24^ Kardorff *et al*.,^25^ Pustišek *et al*.^28^ and Staab *et al*.^35^ all seem to decrease disease severity assessed by SCORing Atopic Dermatitis (SCORAD) and, with the exception of Kardorff *et al*. and the participants aged 13–18 years in the trial reported by Staab *et al*., exceeded the published SCORAD MCID of 8·7.^41^ While all of these studies were set in secondary care and examined interventions for children with eczema, the interventions were different in their nature/intensity (skin model,^25^ 2‐h workshop/education programme,^24^, ^28^ 2‐day education programme,^22^ six 2‐h education sessions),^35^ comparator groups (usual care,^24^, ^25^, ^28^, ^35^ booklet)^22^ and had different durations of follow‐up (from 6 weeks to 12 months).

### Risk of bias

**Figure 3:**
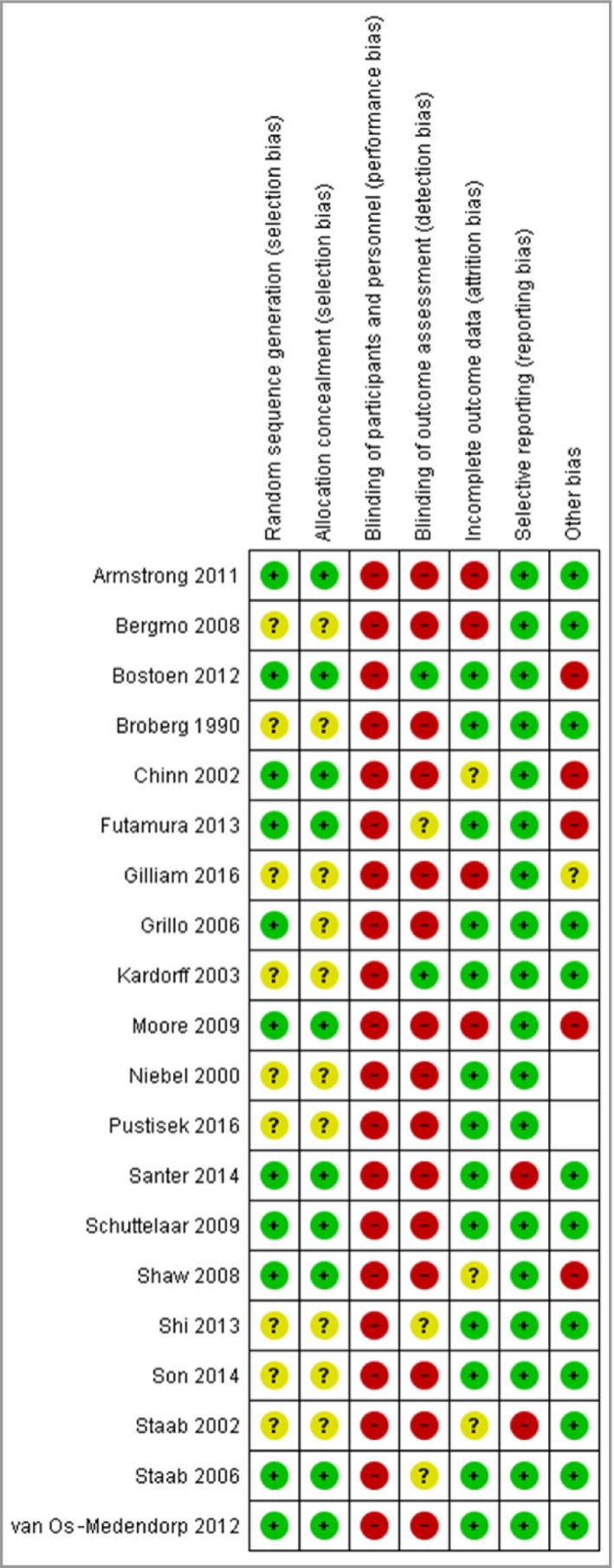
Risk of bias summary. Review authors’ judgements about each risk of bias item for each included study.

Assessments regarding risk of bias in the included studies are summarized graphically in Figure 3. These judgements were difficult to make owing to the generally poor standard of reporting. In trials of self‐management interventions, it is not possible to blind participants to their allocation. Therefore, the majority of trials were graded as ‘high risk’ for this domain.

Six study authors did not state the funding source of their study.^16^, ^17^, ^20^, ^25^, ^26^, ^27^ When specified, the trials were mainly funded by public bodies, with one funded by pharmaceutical companies.^19^ Ten papers declared no conflict of interest,^16^, ^19^, ^22^, ^23^, ^28^, ^29^, ^30^, ^32^, ^35^, ^38^ nine did not state any conflict of interest^17^, ^20^, ^21^, ^25^, ^26^, ^27^, ^31^, ^34^, ^42^ and the one ‘conflict of interest’ declared stated that the study was from an unpublished PhD thesis.^37^

## Discussion

We identified 20 RCTs of interventions that promote self‐management in people with eczema. Most studies had been conducted in Europe or the U.S.A., were based in a hospital setting and targeted children with eczema. The most common type of intervention was face‐to‐face education, but there were wide variations in the nature of these sessions, both in terms of how they were delivered (individually, in groups, or a mixture of both), who delivered them (from one ‘eczema educator’ through to multidisciplinary teams) and their intensity (from 15 min to a total of 24 h). Papers published more recently have focused on interventions delivered via the internet, but again the nature of these interventions varied significantly. All interventions included information on symptom and medication management.

Reporting was generally poor, making it difficult to interpret the findings. Many studies did not specify any criteria for eczema diagnosis. It was often not clear who the ‘caregiver’ was and the methods used to randomize patients were not stated. Interventions or usual care were often described poorly or not at all and it was uncommon for any rationale or formal theory to be given regarding the means by which interventions were expected to effect change. The timing and means of outcome data collection, where specified, were unclear; unpublished or unvalidated measures were frequently employed. Follow‐up was generally short term (12 weeks or less). The absence of any substantial evaluation of cost‐effectiveness is also notable.

We have conducted this review in accordance with current recommendations, have published the review protocol with PROSPERO^9^ and followed PRISMA guidelines for the reporting of reviews evaluating randomized trials.^8^ All screening, data extraction and risk of bias assessments were done by two reviewers independently.

While it is still possible that we may have missed a relevant study, we think this is unlikely because we independently identified relevant studies cited by other related reviews (see below). In the absence of any agreed definition of self‐management, we developed and applied our own criteria based on our reading of the literature. However, given the lack of detail often provided by authors on the content of the different interventions trialled, we consider this to be the safest approach.

Our review complements and extends a number of related reviews that have recently been published, which examine the effect of psychological and educational interventions for eczema on treatment adherence, disease severity and quality of life.^43^, ^44^, ^45^ Our broader remit (all interventions designed to promote self‐management), strong critique of the methods and reporting, and the inclusion of recently published trials will help clinicians, researchers and commissioners better understand what we know about interventions that might help patients with eczema. However, unfortunately, we are in agreement that there is still uncertainty about whether educational interventions are effective in improving quality of life for people with eczema;^46^ most studies of parental education for eczema have been small and of poor quality;^44^ and it is unknown which particular components are clinically effective and cost‐effective in different clinical settings.^47^

We are not alone in noting the ‘preponderance of small, poorly reported and poorly conducted trials’,^47^ which is an issue not confined to just this area of dermatology research. McClean *et al*.^48^ have highlighted the problem specifically with respect to statistical reporting. In keeping with the findings of Alvarez *et al*.,^49^ we found that the standard of reporting was generally better in papers published more recently. However, uncertainty regarding the effectiveness of different interventions to promote self‐management will only be reduced by better designed trials of adequate size reported in line with guidance such as CONSORT^50^ and Statistical Analyses and Methods in the Published Literature.^51^

In addition to improving the reporting of trials (Table 4), researchers should recognize that all interventions to promote self‐management are complex, and their development, description and evaluation should follow an appropriate framework.^52^ Furthermore, interventions should be underpinned by an explicit theory regarding the mechanism of action and preferably accompanied by a process evaluation.^53^ Future studies should seek to evaluate interventions that are pragmatic and tailored to the context and needs of the recipients. In particular, research to date does not reflect the fact that the majority of people with eczema have mild‐to‐moderate disease and are managed in primary care. For example, the needs and likely cost‐effectiveness of an intervention for preschool‐age children is likely to be very different from an intervention for adults with life‐long disease. Despite being recommended by guidelines, the evidence base for written action plans is almost negligible^54^ and, as a potentially low‐cost intervention, warrants particular attention.

**Table 4:** Recommendations to improve conduct and reporting of trials of interventions to promote self‐management in people with eczema

All trials should be prospectively registered, with a trial identifier and a protocol that conforms with CONSORT guidelines published prior to completing participant recruitment Authors should specify which, if any, eczema diagnostic criteria was used and by whom these were administered Studies should clarify which population groups are participating in their trial and at whom the intervention is targeted (children with eczema, caregivers of children with eczema, adults with eczema) and the mechanism by which the authors expect their intervention to work (e.g. improved caregiver knowledge and confidence in use of topical treatments, or improved adherence to treatment in adults with eczema). Studies should state who in the family or otherwise are the main caregivers of children with eczema The type, timing and intensity of the intervention should be described in sufficient detail to enable its replication in clinical practice, observing checklists such as TIDieR^56^ The content of control and comparison groups needs to be described in detail, even if the comparison group is ‘usual care’ because this will vary between settings and countries Primary outcomes within studies need to be specified. Studies should be adequately powered in relation to this. Key outcomes need to be appropriate and relevant to adults and children with eczema and/or their caregivers Outcomes should include core outcomes (symptoms, signs, quality of life, long‐term control) as per Harmonizing Outcomes Measures for Eczema (HOME) recommendations, to enable comparisons across studies and the combination of data in future systematic reviews The timing and method of collection of all outcomes should be stated To reduce detection bias, researchers should give serious consideration to collection of outcomes by an observer blinded to allocation All trials should include an economic evaluation and where appropriate, nested qualitative work and/or a process evaluation

TIDieR, template for intervention description and replication.

While our search and focus was on RCT evidence, the lack of reference to, or use of, qualitative methods in intervention development and evaluation was stark. One encouraging exception to this was the pilot trial by Santer *et al*.,^29^ whose study was supported by both a strong theoretical framework (PRECEDE‐PROCEED) and prior qualitative research. Future trials should also include robust evaluations of the cost‐effectiveness of interventions.

What should clinicians draw from this review? Both internet‐based and face‐to‐face approaches probably improve self‐management and outcomes for patients, but the optimum means of delivering support in a cost‐effective way has yet to be determined.

To return to our original questions, a mixture of different interventions that might promote self‐management have been evaluated and there is evidence that some may be clinically effective. However, it is unknown which components of these interventions (e.g. patient–clinician relationship, use of written action plan) are the most important and cost‐effectiveness has yet to be determined.

## Author contributions

M.J.R. proposed the review question, obtained funding, wrote the protocol, helped develop the literature search, read all of the full‐text articles, designed the data‐extraction tool, undertook data extraction/risk of bias assessments for all included studies, drafted the content of the paper, reworked the first draft and produced the final version, and reworked the response to peer review comments/the manuscript. A.J.L.K. searched the wider literature, researched definitions of self‐management, developed and ran the literature search strategy, deduplicated, applied eligibility criteria and screened 1651 papers, extracted data/undertook risk of bias assessments of 18 of the included studies, drafted the first version of the paper and commented on subsequent redrafts and drafted an initial response to peer review comments. A.L.H. provided methodological expertise and hands‐on guidance in developing, running and importing the results of the literature search strategy as well as the approach to data extraction, interpretation and reporting and contributed to the structure/commented on all versions of the paper. E.L.R. updated the literature search, deduplicated and screened 244 papers, extracted data/undertook risk of bias assessments of two of the included studies, and commented on drafts of the paper. A.W. second screened 1651 papers, translated the two German language papers and commented on drafts of the paper and created the Forest plots.

## Supporting information


**Appendix S1.** Search strategy employed in MEDLINE, MEDLINE in process, Embase and CINAHL databases.
**Appendix S2.** Eczema severity outcomes by study and time point.
**Appendix S3.** Quality‐of‐life outcomes by study and time point.
