# Supplementary material for: Systematic review of self‐management interventions for people with eczema
Source: Br J Dermatol. 2017 Aug 2;177(3):719–34. doi: 10.1111/bjd.15601 (PMC5637890; doi:10.1111/bjd.15601)
Supplement: Supplementary file 1 — Appendix S1. Search strategy employed in MEDLINE, MEDLINE in process, Embase and CINAHL databases. Appendix S2. Eczema severity outcomes by study and time point. Appendix S3. Quality‐of‐life outcomes by study and time point. [file BJD-177-719-s001.docx]

# Appendix 1: Search strategy employed in Medline, Medline in process, Embase and CINAHL databases

1 Eczema/ (8849)

2 Eczema.tw. (11634)

3 atopic eczema.tw. (2183)

4 Dermatitis, Atopic/ (14757)

5 Atopic dermatitis.tw. (12765)

6 1 or 2 or 3 or 4 or 5 (30312)

7 Self Care/ (24461)

8 education.tw. (272699)

9 Patient Education as Topic/ (71646)

10 action plan*.tw. (3647)

11 treatment plan*.tw. (33113)

12 management plan*.tw. (3850)

13 care plan*.tw. (8410)

14 self care.tw. (9896)

15 self management.tw. (7966)

16 individuali#ed plan*.tw. (180)

17 7 or 8 or 9 or 10 or 11 or 12 or 13 or 14 or 15 or 16 (393080)

18. 6 AND 17 (2301)

# Appendix 2: Eczema severity outcomes by study and time point

I = intervention – Santer et al (Iw=web only; Iwh=web plus healthcare professional) and Niebel et al (Id = direct parent education; Iv = video education) had two intervention groups; C=control; n=number of participants; m=mean; SD=standard deviation; - denotes not reported/available

# Appendix 3: QualIty of life outcomes by study and time point

I = intervention – Santer et al (Iw=web only; Iwh=web plus healthcare professional) and Niebel et al (Id = direct parent education; Iv = video education) had two intervention groups; C=control; n=number of participants; m=mean; SD=standard deviation; - denotes not reported/available
